# Optimization of Actuator Stiffness and Actuation Timing of a Passive Ankle Exoskeleton: A Case Study Using a Musculoskeletal Modeling Approach

**DOI:** 10.3390/biomimetics11010002

**Published:** 2025-12-20

**Authors:** Jania Williams, Cody P. Anderson, Arash Mohammadzadeh Gonabadi, Farahnaz Fallahtafti, Sara A. Myers, Hafizur Rahman

**Affiliations:** 1 Department of Biomechanics and Center for Research in Human Movement Variability, University of Nebraska at Omaha, Omaha, NE 68182, USA; 2 School of Health and Kinesiology, University of Nebraska at Omaha, Omaha, NE 68182, USA; 3 Institute for Rehabilitation Science and Engineering, Madonna Rehabilitation Hospitals, Lincoln, NE 68506, USA; 4 Department of Surgery and Research Service, Nebraska-Western Iowa Veterans Affairs Medical Center, Omaha, NE 68105, USA; 5 School of Podiatric Medicine, University of Texas Rio Grande Valley, Harlingen, TX 78550, USA; 6 College of Engineering and Computer Science, University of Texas Rio Grande Valley, Edinburg, TX 78539, USA

**Keywords:** musculoskeletal modeling and simulation, passive ankle exoskeleton, actuator stiffness, actuation timing, energy expenditure

## Abstract

Objective: A modeling and simulation tool, OpenSim, was used to determine the optimal relationship between actuator stiffness and actuation timing of a passive ankle exoskeleton for reducing metabolic costs during walking. We hypothesized that the absolute minimum in total metabolic cost would exist at an actuation timing of 15% of stance and at a spring stiffness of 7.5 kN/m. We also hypothesized that a local minimum in total metabolic cost would exist at an actuation timing of 50% of stance. Methods: Bilateral kinematics and kinetics data were collected on a healthy male walking overground wearing his regular tennis shoe. The passive ankle exoskeleton geometry and the spring actuator were integrated into the OpenSim model. Simulations were performed for every combination of 25 spring stiffnesses ranging from 5.5 kN/m to 17.5 kN/m (increments of 0.5 kN/m) and 10 actuation timings ranging from 15% to 60% of stance (increments of 5%). Total energy expenditure was calculated as the sum of the energy expenditure of all the muscles in the model. Results: The greatest reduction in energy consumption (−2.67%) was observed at an actuation timing of approximately 15% of the stance phase with a spring stiffness of ~5.5 kN/m. A quadratic relationship between spring stiffness and energy consumption was identified (R^2^ = 0.99), with an optimal stiffness of approximately 5.5 kN/m minimizing the energy cost. Conclusions: Our findings suggest that OpenSim effectively predicts optimal exoskeleton parameters, supporting personalized assistance to improve energy efficiency and rehabilitation outcomes.

## 1. Introduction

Exoskeletons augment or supplement muscle force and power to improve walking in older adults, clinical populations, and military service members [1]. In a passive exoskeleton, muscle force and power are usually supplemented by a spring and clutch system that stores and releases energy [2]. Since muscles function similarly to springs, the proper storage and release of energy as in a spring, make locomotion more energy-efficient and can assist lower limb joints during physical activity [2]. Spring provides torque and power, which typically decreases the required muscle and tendon contribution surrounding the joint [3], known as the substitution paradigm. Many studies focus on the ankle joint because the power generated at this joint during push-off is the largest among the other leg joints [4] and has the potential to provide the greatest overall reduction in metabolic cost. Assistance at the ankle is especially beneficial for individuals with mobility impairments, such as those recovering from strokes, living with cerebral palsy, or experiencing age-related musculoskeletal decline.

The overall effectiveness of a passive exoskeleton reducing metabolic costs depends on several factors, including device weight, spring stiffness, and actuation timing. Spring stiffness determines the force provided by the exoskeleton, thereby influencing the levels of muscle activation. The optimal stiffness would minimize muscle activity and reduce the required user effort [5]. Additionally, appropriate stiffness should support natural movement patterns of the ankle [6]. Springs also enhance overall performance due to their ability to complement the natural energy absorption of the soft tissues [7]. The ability of the spring to contribute torque and power during the push-off phase of gait depends on the assistance timing [4]. However, the optimal relationship between actuator stiffness and actuation timing in passive ankle exoskeletons remains unclear, particularly when the goal is to reduce metabolic cost. Our study seeks to address this knowledge gap.

Controlling the timing of spring actuation enables manipulation of the force generated by the spring. The stiffer the spring, the greater the force produced with the same amount of displacement. Choosing the optimal actuation timing is crucial for maximize exoskeleton performance, user experience, and system control [8]. Previous research has found that the optimal actuation timing is near 15% of the stance phase [9]. This timing led to a 21% reduction in metabolic cost during the gait cycle for ten healthy female subjects wearing a powered ankle exoskeleton [9]. However, the optimal actuation timing is individualized and can only be determined by testing several actuation timings for each individual [10]. Exoskeletons have the most potential impact in clinical populations with limited walking ability. A previous study used a passive exoskeleton to support the knee joint during the stance phase of gait for an individual with cerebral palsy [11]. This exoskeleton provided a reduction in hip joint angle and decreased the muscle coactivation index on both the right and left sides of the body. An elastic ankle exoskeleton worn by 11 participants during treadmill walking at various speeds and rotational stiffness observed a reduction in metabolic cost and soleus muscle activity [12]. However, identifying the optimal actuation timing through experimental study is difficult for clinical populations because the process is strenuous and time-consuming, which limits the number of timings and assistance levels that can be tested. Therefore, developing a method to estimate optimal timing could significantly enhance the benefits of exoskeletons for these populations.

Musculoskeletal modeling and simulation have been utilized to understand the complex biomechanics of the ankle and musculotendon dynamics during walking [13,14,15,16]. Modeling enables quick and efficient determination of optimal design parameters before experimental testing. In our previous work, we compared musculoskeletal and joint-space methods for estimating the time profile of metabolic rate during walking. Our results showed that musculoskeletal simulations with muscle-based energetics can accurately track changes in stride-average metabolic costs across walking conditions, highlighting the ability of modeling software to estimate muscle energetics under different movement conditions [17]. OpenSim is a modeling software that simulates, analyzes, and models biomechanics based on initial conditions provided to the model [18]. Although OpenSim software can be computationally expensive and assumes simplified muscle-tendon dynamics and joint constraints that do not capture complex physiological behavior, it can still estimate the overall metabolic cost using built-in cost models that calculate the energy consumption of the muscles within the simulation [5]. Understanding how exoskeletons influence the overall metabolic costs can help identify optimal assistance and timing parameters.

Therefore, the primary goal of this study was to establish a proof of concept that OpenSim simulation modeling can be used to optimize passive ankle exoskeleton stiffness and actuation timing using minimal baseline data, providing an approach useful for clinical populations where extensive testing is not feasible. This study utilized OpenSim to determine the optimal relationship between spring stiffness and actuation timing of a passive ankle exoskeleton for reducing metabolic costs during walking. For this case study, simulations were performed with varying spring stiffness and actuation timings using experimental gait data collected from a healthy male subject. We hypothesized that the absolute minimum metabolic cost would occur at an actuation timing of 15% stance and spring stiffness of 7.5 kN/m. We also hypothesized that a local minimum in metabolic cost exists at an actuation timing of approximately 50% of stance, corresponding to the phase when the center of mass is descending.

## 2. Materials and Methods

### 2.1. Subject

A young, healthy male (age 29 years, height 1.86 m, weight 87.6 kg) was recruited to participate in this case study. The subject was free of musculoskeletal diseases, acute injuries, and gait abnormalities. Written informed consent was obtained from the subject before participation, and all procedures were approved by the University of Nebraska Medical Center Institutional Review Board in accordance with the Declaration of Helsinki.

### 2.2. Experimental Data Collection

Bilateral kinematics and kinetics were collected at the University of Nebraska at Omaha’s Biomechanics Research Building, following the methods described by Rahman [19] and Leutzinger [20]. Reflective markers were adhered to anatomical locations on the subject’s lower limbs, adapted from the marker configurations of Vaughan [21] and Nigg [22]. Kinematic data were captured using 14 high-speed infrared cameras (100 Hz, Motion Analysis Corporation, Rohnert Park, CA, USA), and kinetics were collected from force plates mounted level with the ground (1000 Hz, AMTI, Watertown, MA, USA). The subject walked wearing regular tennis shoe across a 10-m pathway over the force plates until 10 valid foot contacts (5 left and 5 right) were recorded. A foot contact was included in the analysis if a single foot made complete contact with a force plate and there was no evidence of additional forces on the force plate.

### 2.3. Exoskeleton Footwear in OpenSim

**Figure 1:**
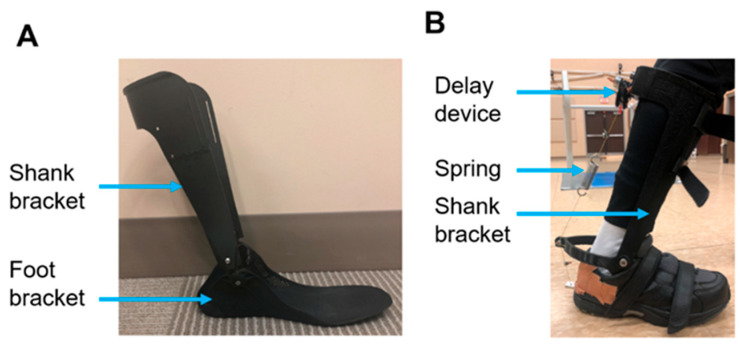
(**A**) The passive ankle exoskeleton comprises a shank bracket and a foot bracket. (**B**) An exchangeable spring was attached to the posterior aspect of the foot bracket. A gravity-actuated delay device was attached to this spring on the posterior–superior aspect of the shank bracket. This device uses gravitational forces to control the timing of mechanical actions, such as the transition between the stance and swing phases of gait.

The passive ankle exoskeleton designed for this project consisted of a carbon fiber foot bracket and a posterior shank bracket (Figure 1A). The foot bracket was designed to be inserted under the insoles of standard tennis shoes. The hinge joint formed between the foot and shank brackets was aligned with the medial and lateral malleoli of the ankle. An exchangeable spring was inserted on the posterior aspect of the foot bracket and attached via a cable to a gravity-actuated delay device on the posterior–superior aspect of the shank bracket (Figure 1B). The gravity-actuated delay device was composed of a rotational wheel with a weighted arm and two face plates that surround the rotational wheel, which were designed to allow for 90 degrees of rotation. A cable was attached to the wheel and the spring. Therefore, pulling on the cable lifts the weighted arm upwards. Energy is not loaded into the spring until the weighted arm reaches its maximum range of motion. This system could effectively delay the onset of energy loading into the spring. The weighted arm is passively reset after the energy is released from the spring by the force of gravity on the weighted arm. The weighted arm of the gravity-actuated delay device could be tuned to different time points of the gait cycle when energy is loaded into the spring (i.e., actuation timing). The passive ankle exoskeleton was designed to store energy in the spring during the early to mid-stance phase and to release the elastic energy in parallel with the posterior calf muscles during toe-off, assisting with plantar flexion and push-off.

**Figure 2:**
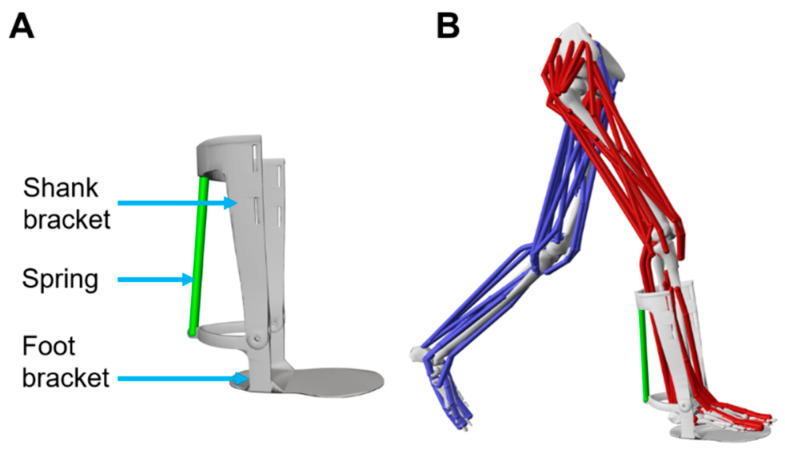
(**A**) The OpenSim passive ankle exoskeleton model comprises a shank bracket, a foot bracket, and a spring actuator. The programmed spring actuator allows spring stiffness to be adjusted. (**B**) OpenSim software integrated the passive ankle exoskeleton model into the pre-existing musculoskeletal model.

To integrate the geometry of the passive ankle exoskeleton into OpenSim, stereolithography (STL) files of the foot bracket and shank bracket were generated with a 3D laser scanner. The foot and shank brackets were programmatically added to the body of a generic “Full Body Musculoskeletal Model” [23], with their respective STL files associated as mesh objects (Figure 2). The mass of the carbon fiber foot and shank brackets was measured with a scale and incorporated programmatically into their respective body set objects. A cylinder body was programmatically added to the body set within their respective local coordinate systems for both the foot and shank brackets. The cylinder bodies were oriented with the axis of rotation of the foot and shank brackets, and a rigid joint was formed between the respective cylinder bodies and the foot and shank brackets [13,24]. The cylinder bodies of the foot and shank brackets were aligned to be on the same axis of rotation, and a hinge joint was programmatically formed between the cylinder bodies, which allowed the foot and shank bracket to hinge about the intended axis of rotation. After aligning the axis of rotation of the exoskeleton with the lateral and medial malleoli of the full-body model, a rigid joint was defined between the shank bracket and the tibia of the full-body model. Furthermore, a linear constraint was applied to the exoskeleton’s hinge joint to ensure its angle always matched that of the ankle joint.

A spring actuator was incorporated into the exoskeleton model to simulate the combined effects of the spring and the actuation delay device. The spring actuator was positioned on the superior-posterior aspect of the shank bracket and the posterior aspect of the foot bracket. The spring stiffnesses chosen for this simulation study were 5.5 kN/m–17.5 kN/m, based on the spring stiffnesses chosen by Collins et al. for a similar passive ankle exoskeleton device [25]. These spring stiffnesses were simulated by modifying the stiffness property of the spring actuator for each simulation. The actuation timings chosen for this study were between 15% and 60% of stance, which approximately corresponds to when the foot is flat after heel strike [25] and when the center of mass is at its peak vertical displacement [26], respectively. Different actuation timings were simulated by programmatically adjusting the length of the spring actuator; longer lengths produced later actuation timings, while shorter lengths resulted in earlier actuation timings. Since the exoskeleton dimensions were known and the exoskeleton angle was constrained in OpenSim to match the sagittal plane ankle angle, determining the resting length of the spring to align with a specific actuation timing involved solving for the spring length using trigonometry. The ankle kinematics were calculated before running the simulations, and these kinematics were used to determine the resting spring lengths that corresponded to specific percentages of stance.

### 2.4. Musculoskeletal Modeling and Simulation in OpenSim

All simulations were performed in OpenSim version 4.0 [18], as previously described [15,16,23]. Briefly, the anthropometry of the entire body model was matched to the subject using the “Scale” tool in OpenSim, which matched the positions of the reflective markers on the subject to the virtual marker set to program the entire body model. For each foot contact, the marker positions were used as input for the “Inverse Kinematics” tool, which minimizes the errors between the experimental marker trajectories and those of the virtual markers on the scaled model to obtain joint angles. The joint angles obtained from the “Inverse Kinematics” tool were input for the “Residual Reduction Algorithm” tool, minimizing errors between the ground reaction forces and the calculated residuals. The kinematics from the “Residual Reduction Algorithm” tool were used as input for the “Computed Muscle Control” tool, which combines proportional–derivative control with static optimization. The metabolic probe was programmed into the full body model and used to estimate the rate of muscle energy expenditure for each muscle, by considering factors such as activation heat rate, maintenance heat rate, shortening/lengthening heat rate, and mechanical work rate of the muscles in the model [27,28,29]. A similar implementation of these Umberger-based metabolic equations in an OpenSim musculoskeletal framework has been used previously to estimate the time profile of metabolic rate during walking and to compare musculoskeletal and joint-space estimation methods [17]. For each simulation, the model was equipped with bilateral exoskeletons; however, actuation was only activated for the limb in contact with the foot. For each foot contact, the simulations were run from 15% of stance (i.e., foot flat on the ground) to the maximum plantarflexion angle after toe-off. Using this timing ensured only one activation timing of the spring actuator, eliminating the influence of the swing phase. Muscle energy expenditure was derived from the metabolic probes for the lower-extremity muscles ipsilateral to the foot contact. The muscles that were included to calculate muscle energy expenditure are listed in Appendix A (Table A1) [23]. Simulations were performed for every combination of 25 spring stiffnesses ranging from 5.5 kN/m to 17.5 kN/m (increments of 0.5 kN/m) and 10 actuation timings ranging from 15% to 60% of stance (increments of 5%), which resulted in 250 exoskeleton settings that were tested for each of the 10-foot contacts (2500 simulations total). A walking simulation video featuring a passive ankle exoskeleton simulated in OpenSim is provided as supplementary material.

### 2.5. Data Analysis

The “Computed Muscle Control” tool exports the metabolic rate of each muscle as an individual time series. The integral of each muscle’s metabolic rate time series was computed to determine its energy expenditure. Total energy expenditure was calculated as the sum of muscle energy expenditures and was reported in Joules per kilogram (J/Kg). Only muscles on the ipsilateral side of the model with respect to the foot contact were summed to avoid contributions from muscles contributing to the contralateral swing phase. The data were expressed as the mean total energy expenditure of all 10-foot contacts (left and right). The changes in energy expenditure were determined by subtracting the baseline walking energy expenditure (without the exoskeleton) from that recorded for each exoskeleton setting (combination of spring stiffness and actuation timing). Negative values indicate that the subject used less energy walking with the exoskeleton, while positive values indicate increased energy consumption. Data processing and analyses were performed using custom codes in MATLAB 2019b (The MathWorks Inc., Natick, MA, USA) and Python 3.8 (Python Software Foundation, Wilmington, DE, USA).

### 2.6. Statistics

A one-way analysis of variance was used to test for differences in the means of local minimums in energy expenditure when using the exoskeleton compared to using the exoskeleton with no actuation, and when not using the exoskeleton. Tukey’s test was employed for post hoc analysis. All data were checked for normality with the Shapiro–Wilk test before conducting parametric tests. Significance was set at *p* < 0.05. All statistical analyses were performed in GraphPad Prism (GraphPad Prism Software, V. 9.0.2, San Diego, CA, USA). Data are presented as means ± standard deviation (STD).

### 2.7. Validation of the OpenSim Exoskeleton Model

**Figure A1:**
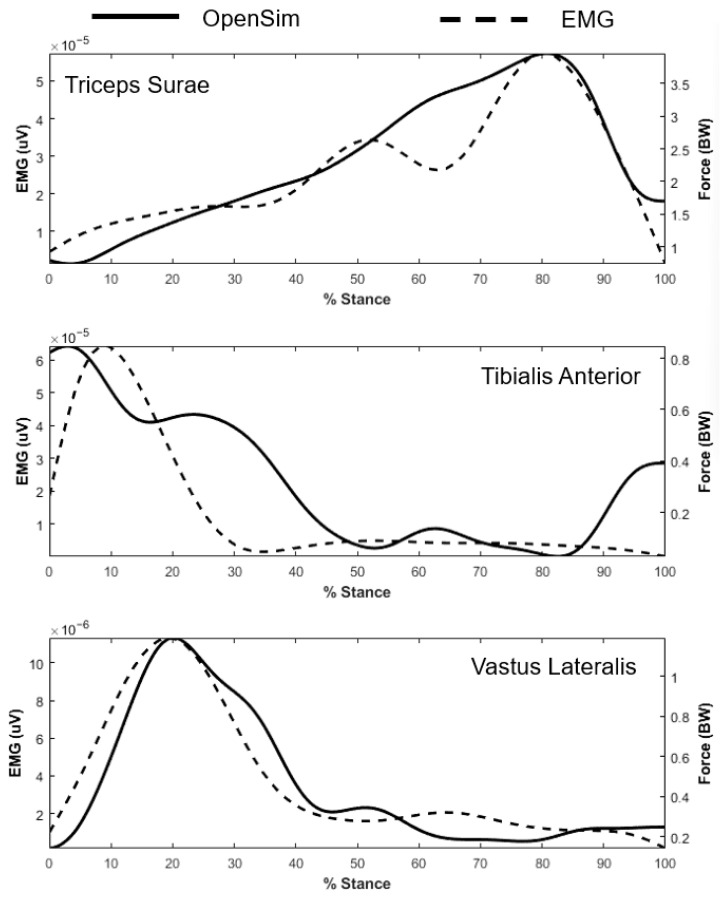
The comparison of experimental data with OpenSim simulation results for the C1 condition (no exoskeleton). The solid line shows the muscle forces estimated by the OpenSim simulation. The dotted line shows the muscle activations recorded by EMG. Muscle forces are expressed in body weight (BW), and muscle activations are expressed in µV.

**Figure A2:**
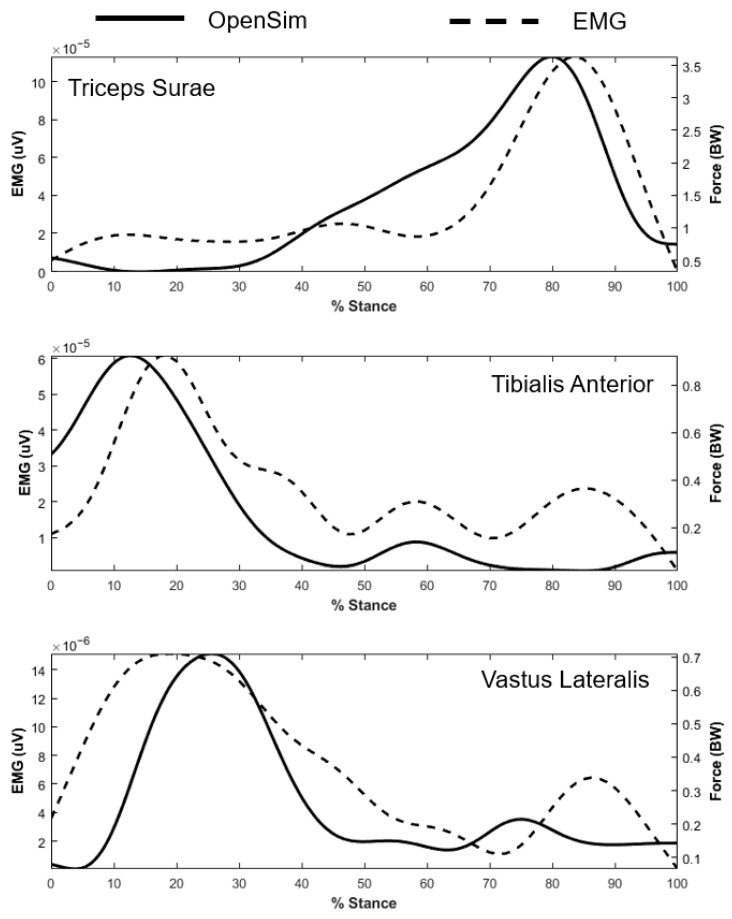
The comparison of experimental data with OpenSim simulation results for the C2 condition (exoskeleton with no actuation, meaning a disengaged spring). The solid line shows the muscle forces estimated by the OpenSim simulation. The dotted line shows the muscle activations recorded by EMG. Muscle forces are expressed in body weight (BW), and muscle activations are expressed in µV.

**Figure A3:**
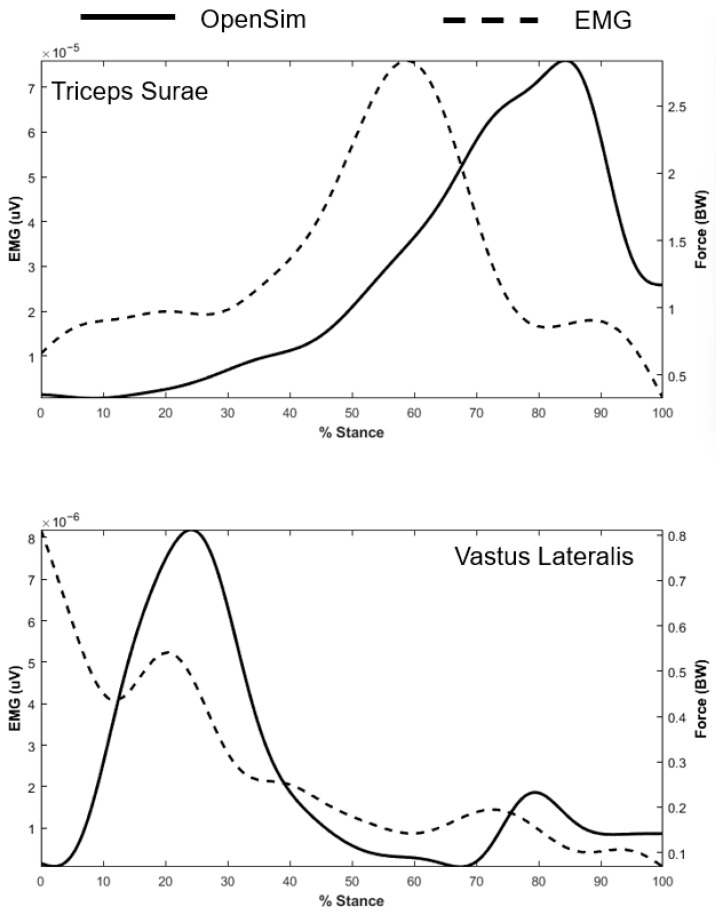
The comparison of experimental data with OpenSim simulation results for the C3 condition (exoskeleton with a 5.6 kN/m spring). The solid line shows the muscle forces estimated by the OpenSim simulation. The dotted line shows the muscle activations recorded by EMG. Muscle forces are expressed in body weight (BW), and muscle activations are expressed in µV.

**Figure A4:**
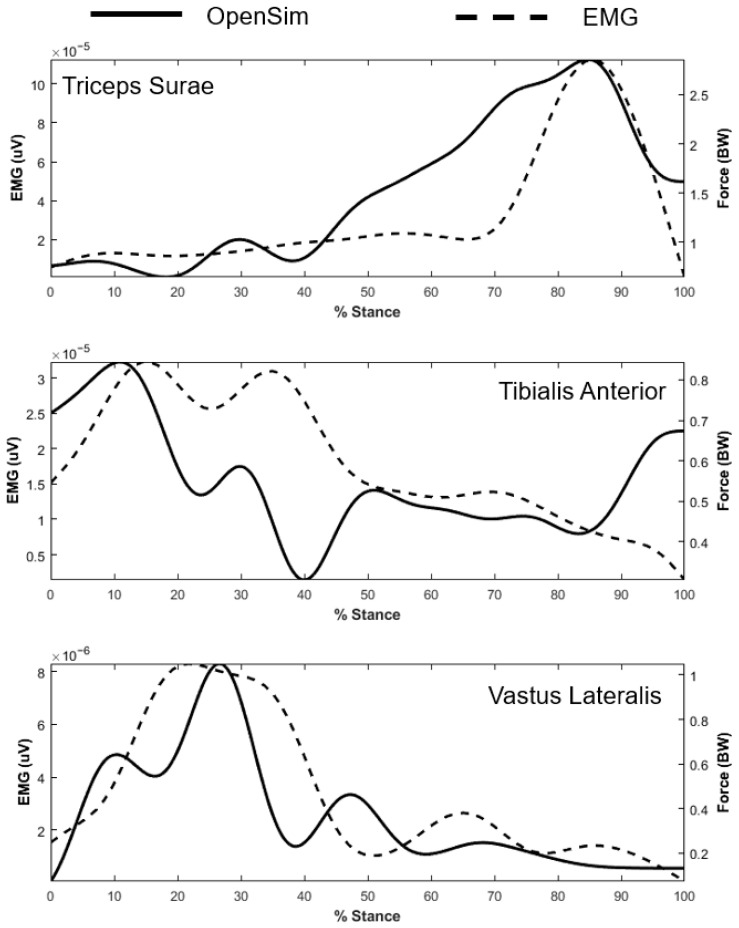
The comparison of experimental data with OpenSim simulation results for the C4 condition (exoskeleton with a 10.5 kN/m spring). The solid line shows the muscle forces estimated by the OpenSim simulation. The dotted line shows the muscle activations recorded by EMG. Muscle forces are expressed in body weight (BW), and muscle activations are expressed in µV.

To validate our exoskeleton model in OpenSim, we collected additional lower extremity kinematics, kinetics, and electromyography (EMG) data as a participant walked while wearing the exoskeleton under different conditions. The muscle forces generated by the OpenSim simulation were compared with EMG activations of four lower extremity muscles to validate the model. The details of the validation procedure are explained in Appendix B (Figure A1, Figure A2, Figure A3 and Figure A4). This validation strategy is consistent with previous work that used an EMG-driven musculoskeletal OpenSim model to estimate metabolic rate time profiles during walking [17].

## 3. Results

### 3.1. Effects of Actuator Stiffness

**Figure 3:**
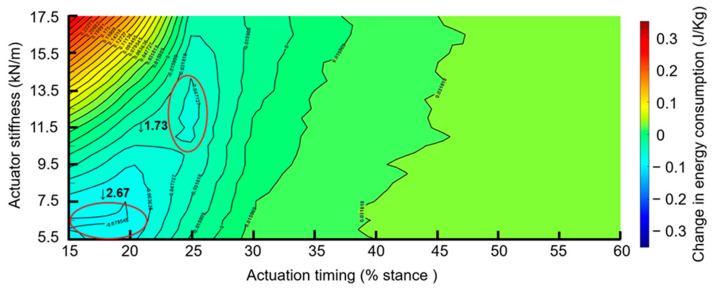
Effects of actuator stiffness and actuation timing on energy consumption during the stance phase. The color illustrates the change in energy consumption relative to walking without exoskeleton footwear. The greatest reduction in energy consumption (−2.67%) was observed at an actuation timing of 15% of the stance phase with a spring stiffness of ~5.5 kN/m. A reduction of 1.73% occurred at an actuation timing of 25% of the stance phase, and a spring stiffness of 12 kN/m. These are indicated by red circles on the graph.

**Figure 4:**
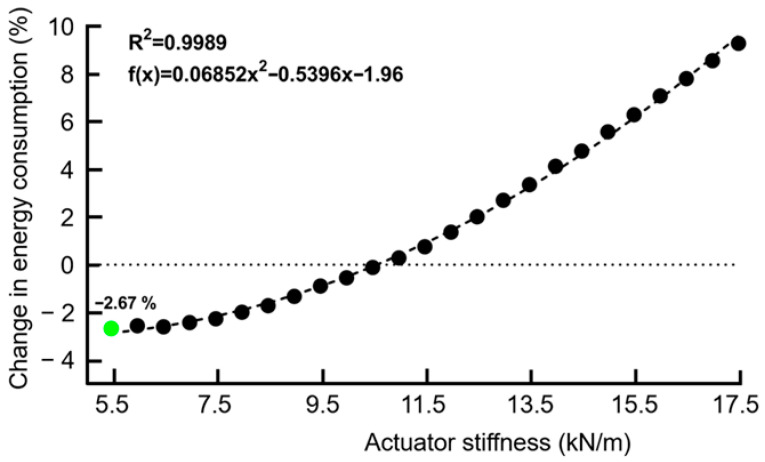
Change in energy consumption during walking at an actuation timing of 15% of the stance phase as actuator stiffness values varied from 5.5 to 17.5 kN/m. A quadratic relationship was observed between spring stiffness and energy consumption (R^2^ = 0.99), with an optimal stiffness of ~5.5 kN/m, which minimizes energy cost. The lowest point on the curve (−2.67%) corresponds to an optimal spring stiffness of approximately 5.5 kN/m, representing the stiffness value that minimizes energy consumption relative to baseline walking.

For the minimum spring stiffness of 5.5 kN/m, the total energy consumption during walking was negative at 15% actuation timing (Figure 3 and Figure 4). This indicates that less energy was expended while wearing the exoskeleton at an actuation timing of 15% of the stance phase compared to the no-actuation condition. This reduction in energy consumption persisted until approximately 27% of the stance phase had elapsed. Beyond this point, as actuation timing increased, total energy consumption exceeded baseline levels, indicating a loss of metabolic efficiency. This trend continued through 60% of the stance phase, though the magnitude of the increase remained relatively stable (Figure 3). Total energy consumption was positive for the maximum spring stiffness of 17.5 kN/m (i.e., higher than baseline) from 15% to 60% at all actuation timings (Figure 3), suggesting that excessively high stiffness introduces resistance that increases metabolic cost.

### 3.2. Effects of Actuation Timing

At an actuation timing of 15% of stance, total energy consumption was negative at lower stiffness values (5.5–10.5 kN/m) and reached a minimum at approximately 5.5 kN/m, corresponding to a maximum reduction of −2.67% in energy consumption (Figure 3 and Figure 4). After a stiffness of 5.8 kN/m, the change in energy consumption was lower than walking without the exoskeleton until ~10.5 kN/m. Beyond a stiffness of ~10.5 kN/m, energy consumption increased and exceeded the baseline, indicating that higher stiffness values decreased metabolic efficiency. Additionally, the effect of stiffness was more pronounced until actuation timings of 30–35% of the stance phase, after which the total energy consumption became relatively invariant to changes in stiffness.

### 3.3. Combining Effects of Actuator Stiffness and Actuation Timing

**Figure 5:**
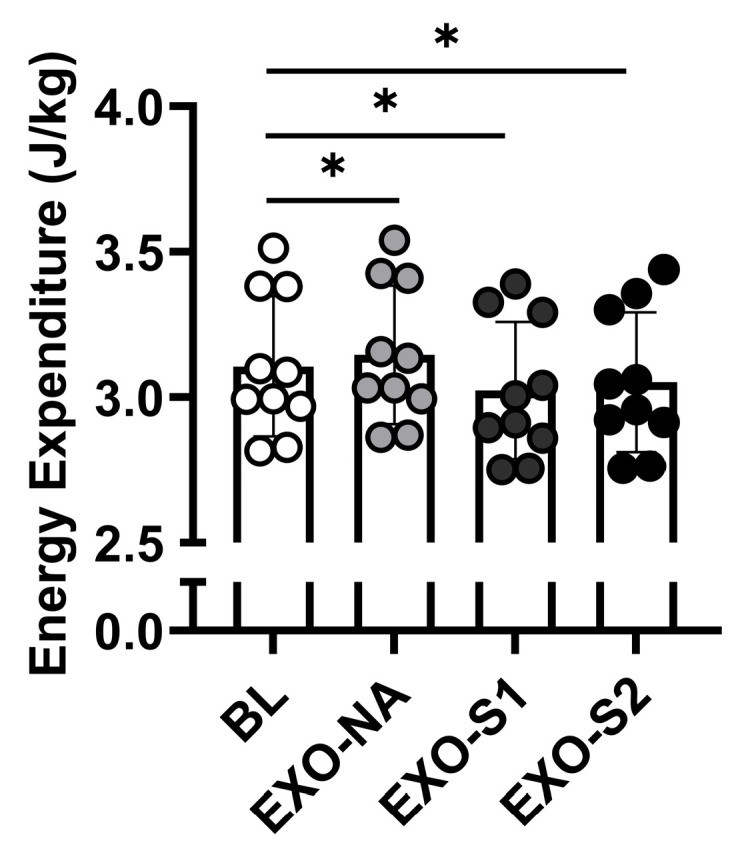
Energy expenditure with different exoskeleton settings for 10-foot contacts. The energy consumed without the exoskeleton at baseline (BL), with the exoskeleton without actuation (EXO-NA), with the exoskeleton at an actuation timing of 15% stance and spring stiffness of 5.5 kN/m (EXO-S1), and with the exoskeleton at an actuation timing of 25% stance and a spring stiffness of 12 kN/m (EXO-S2). * *p* < 0.05 compared to BL.

The energy consumed during walking without the exoskeleton was 3.11 ± 0.24 J/kg (Figure 5). Walking with the exoskeleton without actuation (3.14 ± 0.24 J/kg) had a greater energy consumption than walking without the exoskeleton (*p* < 0.001, Figure 5). The greatest global minimum in energy consumption occurred with the exoskeleton with an actuation timing of 15% and a spring stiffness of 5.5 kN/m (3.02 ± 0.24 J/kg), which was lower than walking without the exoskeleton (*p* < 0.001, Figure 5), and corresponded with a −2.67% reduction in the metabolic cost of walking (Figure 3 and Figure 4). The second greatest minimum in energy consumption occurred with the exoskeleton with an actuation timing of 25% stance and a spring stiffness of 12 kN/m (3.05 ± 0.24 J/kg), which was lower than walking without the exoskeleton (*p* < 0.001, Figure 5), and corresponded with a −1.73% reduction in the metabolic cost of walking (Figure 3 and Figure 4).

## 4. Discussion

This early feasibility study utilized the OpenSim modeling and simulation tool to determine the optimal relationship between actuator stiffness and actuation timing for a passive ankle exoskeleton in a single healthy subject. We hypothesized that the absolute minimum in total metabolic cost would exist at an actuation timing of 15% of stance and at a spring stiffness of 7.5 kN/m based on recent findings [9,25]. We also hypothesized that a local minimum in total metabolic cost would exist at an actuation timing of ~50% of stance due to the center of mass descending at this point in the gait cycle. Bilateral kinematics and kinetics data were collected experimentally and were input into OpenSim. Passive ankle exoskeleton geometry and the spring actuator were integrated into the OpenSim musculoskeletal model. The spring stiffness and actuation timing were varied in the software, and total energy expenditure was estimated. The results of this study align with our first hypothesis, which posits that the absolute minimum in total metabolic cost occurs at an actuation timing of 15% and near a spring stiffness of 7.5 kN/m. Contrary to our second hypothesis, no local minimum in metabolic cost was observed at ~50% of stance. The most likely explanation for this finding is that spring assistance was progressively reduced at later actuation timings, thereby providing negligible assistance during plantarflexion.

### 4.1. Impact of Actuation Timing

The optimal reduction in total energy consumption (−2.67%) was observed at an actuation timing of 15% of the stance phase at a spring stiffness of ~5.5 kN/m. This finding aligns with the results of Collins et al., who utilized an unpowered exoskeleton while walking [25]. However, an additional observation about actuation timing and spring stiffness was made. Interestingly, beyond an actuation timing of about 25%, the passive exoskeleton did not meaningfully reduce total energy consumption, regardless of actuator stiffness. This is likely explained by reduced energy loaded into the spring at later actuation timings. Notably, later actuation timings resulted in less stretching of the spring and less time for energy loading. Therefore, the energy contributed by the spring to plantarflexion at later actuations is likely negligible. To produce a minimum in energy expenditure later in stance, it is likely that much stiffer springs would have been necessary, which may not be feasible in a real ankle exoskeleton. Therefore, in the healthy adult studied, actuation timing during mid-to-late stance may not provide meaningful assistance during the energy-demanding phases of walking, such as push-off [9]. Based on our findings, for the healthy adult studied, the latest activation of the actuator should only occur until 25–30% of stance because after this point, the exoskeleton provides no meaningful reductions in total metabolic cost. In support of these findings, several other studies have found that the optimal actuation timing may be between 25% and 42% for passive ankle exoskeletons [9,30,31].

### 4.2. Impact of Actuator Stiffness

The effects of actuator stiffness were more prominent until an actuation timing of ~25% of stance. At 15% of stance, lower actuator stiffness values resulted in a more substantial reduction in energy consumption. However, as stiffness increased, the total metabolic cost also increased and exceeded the baseline; thus, walking with the exoskeleton became less efficient. The early stance phase of gait occurs between 0% and 25%, where the body absorbs impact forces. Appropriate stiffness can complement ankle mechanics and reduce the metabolic cost during this time [12]. Conversely, if the stiffness is too high, antagonist muscles must work harder to overcome the spring’s resistance [12]. Similar results were also observed in a study investigating the impact of stiff-insole shoes on metabolic cost [32]. In the study, participants walked at three different speeds and insole stiffnesses, and researchers observed a 9.6% increase in energy cost at the typical walking speed, indicating a metabolic rate disadvantage based on increased insole stiffness [32]. Therefore, in our modeling study, lower actuator stiffnesses around an actuation timing of 15%, where the body absorbs impact, may be optimal for decreasing energy consumption in the healthy adult that was studied. This may be attributed to the mechanical interplay between the exoskeleton’s spring properties and the subject’s natural biomechanics. Moderate stiffness allows for efficient energy storage and return, reducing the muscle load. However, excessive stiffness disrupts this balance, requiring the subject’s muscles to exert more effort to overcome the spring’s resistance. This is likely because the natural mechanics of the muscles and tendons dominate energy storage and release during mid-to-late stance, diminishing the impact of the exoskeleton. Energy consumption remained constant until spring stiffness exceeded 10.5 kN/m, suggesting a potential threshold where additional stiffness no longer complements the subject’s natural energy storage and return mechanisms. Future research should explore the role(s) of antagonistic muscles in determining the optimal exoskeleton parameters for individual subjects and patients.

### 4.3. Advantages of Modeling and Parametric Study

The effectiveness of exoskeletons has been previously validated; however, research has shown that the effectiveness of exoskeleton parameters may vary between individuals [33,34]. Therefore, it is essential to understand the relationship between actuation timing and actuator assistance as well as how these factors combine to achieve optimal metabolic efficiency for individual subjects and patients. However, this process is time-consuming and challenging in an experimental setting due to the unlimited combination of potential exoskeleton assistance parameters [31]. Our approach outlined herein utilized OpenSim modeling and simulation tool to sweep through assistance magnitude and timing parameters, identifying the optimal combination that results in the minimum energy consumption for a single subject. Other studies that have used simulations and human subjects have observed similar benefits at actuation timings occurring in early stance [9], optimal spring stiffness for a powered exoskeleton at 6 kN/m [35], and similar energy consumption results to those in this study, further validating the results from this method [25]. Moreover, simulation software enables the exploration of a broader range of design possibilities than physical experiments, providing valuable initial estimates for optimization [1].

### 4.4. Implications and Clinical Applications

This study has possible implications for the individualized design and optimization of passive ankle exoskeletons, particularly for clinical populations. The use of passive ankle exoskeletons is prevalent in populations with walking limitations due to calf insufficiencies, such as patients with peripheral artery disease, where physical demands must be reduced [36]. Additionally, the use of passive ankle exoskeletons can target elderly individuals with impaired plantar flexor muscle strength [37]. Our passive exoskeleton device may enhance push-off during gait, reducing muscle load and lowering metabolic cost when properly optimized. By using OpenSim to identify the optimal combination of actuator stiffness and actuation timing for an individual subject, this study provides a framework to enhance the efficiency of these devices in reducing the metabolic cost of walking. The observed reduction in energy consumption at specific stiffness and timing parameters suggests that personalized exoskeleton settings could improve user comfort and effectiveness, thereby promoting greater adherence to assistive device usage [38]. OpenSim modeling can predict optimal parameters without the need for extensive physical testing. This simplifies the design process, making it less burdensome for patients and reducing the cost and time required for customization. For rehabilitation applications, the findings could guide therapists and engineers in tailoring exoskeletons to individual needs, enabling patients to achieve maximum mobility with minimal effort. Furthermore, integrating optimized exoskeletons into therapy could enhance gait mechanics, reduce fatigue, and improve overall quality of life. Parameters can be further optimized by integrating various tools, such as MATLAB, to evaluate how different exoskeleton parameters affect the user’s gait movement dynamics, such as joint angles [38]. OpenSim can also track and predict future user movement by collecting additional experimental data, helping to accelerate the design and production process.

### 4.5. Limitations

Due to the nature of this parametric case study, a single set of kinematic data from a healthy individual was used to simulate various actuator stiffnesses and actuation timing in OpenSim to determine the optimal exoskeleton design parameters. The data provided by OpenSim may differ if kinematic and electromyography data are directly obtained from experimental testing, where outcome measures are collected in real-time as the subject walks. However, previous literature that experimentally assessed passive exoskeleton settings found marginal impacts on kinematics across the range of spring stiffness chosen for this study [25]. Another limitation is that the muscle properties in the simulations were based on default OpenSim values, which may affect the generalizability of these findings to clinical populations. As shown in previous work comparing joint-space and musculoskeletal metabolic estimations [17], estimates of metabolic cost are method-dependent; therefore, the percentage changes in energy consumption reported here should be interpreted as model-based trends rather than exact subject-specific predictions.

### 4.6. Future Studies

Future studies should expand on these results by collecting data on additional subjects to further validate and determine the reliability of the data from OpenSim simulations. Additionally, future studies should utilize OpenSim to predict optimal exoskeleton settings for multiple subjects and then experimentally validate these settings to confirm the accuracy of the OpenSim predictions. Future work could combine the parametric optimization approach used here with detailed analyses of the metabolic rate time profile, similar to our previous comparison of joint-space and musculoskeletal methods [17], to identify which phases of gait benefit most from a passive ankle exoskeleton. This would enable a statistical analysis to compare the results of laboratory-obtained data to simulation data. Furthermore, because the optimal exoskeleton settings in this study were not experimentally confirmed, future work should validate simulation results against experimental data.

## 5. Conclusions

This study used OpenSim to optimize a personalized actuation timing (~15% stance) and actuator stiffness (5.5–8.0 kN/m) for minimizing metabolic cost with a passive ankle exoskeleton in an individual subject. These findings provide a foundation for personalized exoskeleton design, reducing energy expenditure, and improving gait efficiency. These results align with those of other studies, providing evidence that OpenSim simulations may be a viable tool for predicting optimal exoskeleton parameters for passive ankle exoskeletons. This can directly be applied to aid in designing and developing passive ankle exoskeletons for clinical populations.

## Data Availability

Data will be made available on request.

## Supplementary Materials

The following supporting information can be downloaded at https://www.mdpi.com/article/10.3390/biomimetics11010002/s1: Video S1: Walking simulation with passive ankle exoskeleton in OpenSim.

## Appendix A

**Table A1:** List of muscles included to calculate muscle energy expenditure [23].

Muscle Name
Adductor brevis
Adductor longus
Adductor magnus (distal)
Adductor magnus (ischial)
Adductor magnus (middle)
Adductor magnus (proximal)
Biceps femoris long head
Biceps femoris short head
Extensor digitorum longus
Extensor hallucis longus
Flexor digitorum longus
Flexor hallucis longus
Gastrocnemius lateral head
Gastrocnemius medial head
Gluteus maximus (superior)
Gluteus maximus (middle)
Gluteus maximus (inferior)
Gluteus medius (anterior)
Gluteus medius (middle)
Gluteus medius (posterior)
Gluteus minimus (anterior)
Gluteus minimus (middle)
Gluteus minimus (posterior)
Gracilis
Iliacus
Peroneus brevis
Peroneus longus
Piriformis
Psoas
Rectus femoris
Sartorius
Semimembranosus
Semitendinosus
Soleus
Tensor fascia latae
Tibialis anterior
Tibialis posterior
Vastus intermedius
Vastus lateralis
Vastus medialis

## Appendix B. Validation of the OpenSim Exoskeleton Model

To validate our exoskeleton model, we compared the muscle forces generated by the OpenSim simulation with electromyography (EMG) data collected from several lower extremity muscles as a person walked while wearing the exoskeleton at different stiffness levels. For the experimental test, a participant completed walking trials across four conditions. The conditions were: (i) C1: no exoskeleton, (ii) C2: exoskeleton with no actuation, meaning a disengaged spring, (iii) C3: exoskeleton with a 5.6 kN/m spring, and (iv) C4: exoskeleton with a 10.5 kN/m spring. The participant walked on overground force plates level with the ground (AMTI, Watertown, MA) for each condition until one full contact was made. A full contact was defined as the entire foot touching the force plate with no other forces acting on it during stance. Kinematic data were captured using an array of high-speed infrared cameras (Motion Analysis Corporation, Rohnert Park, CA, USA). During the experiment, EMG sensors were placed on four muscles: medial gastrocnemius, soleus, tibialis anterior, and vastus lateralis. EMG data were collected at a sampling rate of 1000 Hz during overground walking trials. The EMG signals were then filtered, integrated, and rectified.

The kinematic and kinetic data for each condition served as inputs for the OpenSim simulations. The simulation process was consistent with the experimental workflow described in Section 2.4. Briefly, joint angles were calculated using the kinematic data and the “Inverse Kinematics” tool. The inverse kinematics and kinetics data were then used with the “Computed Muscle Control” tool to estimate forces in the lower extremity muscles. All forces were normalized to body weight. The force time series for the medial gastrocnemius, soleus, tibialis anterior, and vastus lateralis were exported and trimmed from heel-strike to toe-off to match the corresponding EMG time series for each muscle. OpenSim force and EMG data were plotted together to visually verify the agreement between in vivo EMG and OpenSim force estimates. For simplicity, the EMG and forces of the medial gastrocnemius and soleus were summed to represent the overall EMG and force of the triceps surae muscle group. Our analysis shows strong agreement between EMG activations and OpenSim force estimates across all muscle groups and conditions (Figure A1, Figure A2, Figure A3 and Figure A4). Therefore, our validation sub-analysis supports the conclusion that the OpenSim simulations used to estimate muscle forces with the ankle exoskeleton align well with in vivo EMG activations.

## References

[1] Siviy C., Baker L.M., Quinlivan B.T., Porciuncula F., Swaminathan K., Awad L.N., Walsh C.J. (2022). Opportunities and challenges in the development of exoskeletons for locomotor assistance. Nat. Biomed. Eng..

[2] Gorgey A.S., Sumrell R., Goetz L.L. (2019). Exoskeletal Assisted Rehabilitation After Spinal Cord Injury. Atlas of Orthoses and Assistive Devices.

[3] Allen S.P., Grabowski A.M. (2024). The spring stiffness profile within a passive, full-leg exoskeleton affects lower-limb joint mechanics while hopping. R. Soc. Open Sci..

[4] Bougrinat Y., Achiche S., Raison M. (2019). Design and development of a lightweight ankle exoskeleton for human walking augmentation. Mechatronics.

[5] Seth A., Hicks J.L., Uchida T.K., Habib A., Dembia C.L., Dunne J.J., Ong C.F., Demers M.S., Rajagopal A., Millard M. (2018). OpenSim: Simulating musculoskeletal dynamics and neuromuscular control to study human and animal movement. PLoS Comput. Biol..

[6] Totah D., Menon M., Jones-Hershinow C., Barton K., Gates D.H. (2019). The impact of ankle-foot orthosis stiffness on gait: A systematic literature review. Gait Posture.

[7] Barrutia W.S., Yumiceva A., Thompson M.L., Ferris D.P. (2024). Soft tissue can absorb surprising amounts of energy during knee exoskeleton use. J. R. Soc. Interface.

[8] Peng X., Acosta-Sojo Y., Wu M.I., Stirling L. (2022). Actuation Timing Perception of a Powered Ankle Exoskeleton and Its Associated Ankle Angle Changes During Walking. IEEE Trans. Neural Syst. Rehabil. Eng..

[9] Galle S., Malcolm P., Collins S.H., De Clercq D. (2017). Reducing the metabolic cost of walking with an ankle exoskeleton: Interaction between actuation timing and power. J. Neuroeng. Rehabil..

[10] Kim J., Quinlivan B.T., Deprey L.-A., Revi D.A., Eckert-Erdheim A., Murphy P., Orzel D., Walsh C.J. (2022). Reducing the energy cost of walking with low assistance levels through optimized hip flexion assistance from a soft exosuit. Sci. Rep..

[11] Kennard M., Kadone H., Shimizu Y., Suzuki K. (2022). Passive exoskeleton with gait-based knee joint support for individuals with cerebral palsy. Sensors.

[12] Nuckols R.W., Nuckols R.W., Nuckols R.W., Sawicki G.S., Sawicki G.S. (2020). Impact of elastic ankle exoskeleton stiffness on neuromechanics and energetics of human walking across multiple speeds. J. Neuroeng. Rehabil..

[13] Arch E.S., Stanhope S.J., Higginson J.S. (2016). Passive-dynamic ankle-foot orthosis replicates soleus but not gastrocnemius muscle function during stance in gait: Insights for orthosis prescription. Prosthet. Orthot. Int..

[14] Choi H., Bjornson K., Fatone S., Steele K.M. (2016). Using musculoskeletal modeling to evaluate the effect of ankle foot orthosis tuning on musculotendon dynamics: A case study. Disabil. Rehabil. Assist. Technol..

[15] Rahman H., Anderson C.P., Pipinos I.I., Johanning J.M., Casale G.P., Dong J., DeSpiegelaere H., Hassan M., Myers S.A. (2022). Muscle forces and power are significantly reduced during walking in patients with peripheral artery disease. J. Biomech..

[16] Anderson C.P., Pipinos I.I., Johanning J.M., Myers S.A., Rahman H. (2024). Effects of Supervised Exercise Therapy on Muscle Function During Walking in Patients with Peripheral Artery Disease. Bioengineering.

[17] Gonabadi A.M., Antonellis P., Malcolm P. (2020). Differences between joint-space and musculoskeletal estimations of metabolic rate time profiles. PLoS Comput. Biol..

[18] Delp S.L., Anderson F.C., Arnold A.S., Loan P., Habib A., John C.T., Guendelman E., Thelen D.G. (2007). OpenSim: Open-source software to create and analyze dynamic simulations of movement. IEEE Trans. Biomed. Eng..

[19] Rahman H., Leutzinger T., Hassan M., Schieber M., Koutakis P., Fuglestad M.A., DeSpiegelaere H., Longo G.M., Malcolm P., Johanning J.M. (2024). Peripheral artery disease causes consistent gait irregularities regardless of the location of leg claudication pain. Ann. Phys. Rehabil. Med..

[20] Leutzinger T.J., Koutakis P., Fuglestad M.A., Rahman H., Despiegelaere H., Hassan M., Schieber M., Johanning J.M., Stergiou N., Longo G.M. (2022). Peripheral artery disease affects the function of the legs of claudicating patients in a diffuse manner irrespective of the segment of the arterial tree primarily involved. PLoS ONE.

[21] Vaughan C.L., Davis B.L., O’Connor J.C. (1999). Dynamics of Human Gait.

[22] Nigg B.M., Cole G.K., Nachbauer W. (1993). Effects of arch height of the foot on angular motion of the lower extremities in running. J. Biomech..

[23] Rajagopal A., Dembia C.L., DeMers M.S., Delp D.D., Hicks J.L., Delp S.L. (2016). Full-Body Musculoskeletal Model for Muscle-Driven Simulation of Human Gait. IEEE Trans. Biomed. Eng..

[24] Yamamoto M., Shimatani K., Hasegawa M., Murata T., Kurita Y. (2018). Estimation of knee joint reaction force based on the plantar flexion resistance of an ankle-foot orthosis during gait. J. Phys. Ther. Sci..

[25] Collins S.H., Bruce Wiggin M., Sawicki G.S. (2015). Reducing the energy cost of human walking using an unpowered exoskeleton. Nature.

[26] Lee C.R., Farley C.T. (1998). Determinants of the center of mass trajectory in human walking and running. J. Exp. Biol..

[27] Uchida T.K., Hicks J.L., Dembia C.L., Delp S.L. (2016). Stretching Your Energetic Budget: How Tendon Compliance Affects the Metabolic Cost of Running. PLoS ONE.

[28] Umberger B.R., Gerritsen K.G.M., Martin P.E. (2003). A model of human muscle energy expenditure. Comput. Methods Biomech. Biomed. Eng..

[29] Umberger B.R. (2010). Stance and swing phase costs in human walking. J. R. Soc. Interface.

[30] Choi G., Lee D., Kang I., Young A.J. (2021). Effect of Assistance Timing in Knee Extensor Muscle Activation During Sit-to-Stand Using a Bilateral Robotic Knee Exoskeleton. Annu. Int. Conf. IEEE Eng. Med. Biol. Soc..

[31] Lakmazaheri A., Song S., Vuong B.B., Biskner B., Kado D.M., Collins S.H. (2024). Optimizing exoskeleton assistance to improve walking speed and energy economy for older adults. J. Neuroeng. Rehabil..

[32] Ray S.F., Takahashi K.Z. (2020). Gearing Up the Human Ankle-Foot System to Reduce Energy Cost of Fast Walking. Sci. Rep..

[33] Chen Y., Chen G., Ye J., Fu C., Liang B., Li X. (2023). Learning to Assist Different Wearers in Multitasks: Efficient and Individualized Human-In-the-Loop Adaption Framework for Exoskeleton Robots. IEEE Trans. Robot..

[34] Shushtari M., Foellmer J., Arami A. (2024). Human–exoskeleton interaction portrait. J. Neuroeng. Rehabil..

[35] Magnúsdóttir Í.D. (2020). Simulation of Spring Uses in an Ankle Exoskeleton During Human Gait. Kth Royal Institute of Technology. https://kth.diva-portal.org/smash/record.jsf?pid=diva2%3A1468986&dswid=8940.

[36] Mays R.J., Mays A.A., Mizner R.L. (2019). Efficacy of ankle-foot orthoses on walking ability in peripheral artery disease. Vasc. Med..

[37] Yandell M.B., Tacca J.R., Zelik K.E. (2019). Design of a Low Profile, Unpowered Ankle Exoskeleton That Fits Under Clothes: Overcoming Practical Barriers to Widespread Societal Adoption. IEEE Trans. Neural Syst. Rehabil. Eng..

[38] Hidayah R., Sui D., Wade K.A., Chang B.C., Agrawal S. (2021). Passive knee exoskeletons in functional tasks: Biomechanical effects of a SpringExo coil-spring on squats. Wearable Technol..

